# Beyond the canonical niche: how astrocytes carried neurogenic potential into the brain parenchyma

**DOI:** 10.3389/fcell.2025.1737065

**Published:** 2026-01-23

**Authors:** Marco Fogli, Giulia Nato, Paolo Peretto, Annalisa Buffo, Federico Luzzati

**Affiliations:** 1 Deparment of Life Sciences and Systems Biology, University of Turin, Turin, Italy; 2 Neuroscience Institute Cavalieri Ottolenghi, Orbassano, Italy; 3 Deparment of Neurosciences “Rita Levi Montalcini”, University of Turin, Turin, Italy

**Keywords:** adult neurogenesis, astrocyte diversity, astrocytes reactivity, brain regeneration, glia evolution, neural stem cells, parenchymal neurogenesis, radial glia

## Abstract

The cellular and molecular programs underlying neurogenesis are deeply conserved in metazoans. In vertebrates, neural progenitor and glial lineages converged within the astroglia lineage, which can alternate between stem cell activity and homeostatic states that support neuronal function. In mammals, astroglia migrated into the parenchyma, where they further diversified both between and within regions and specialized in homeostatic support, while only two restricted populations retained neurogenic activity in the ventricular-subventricular (V-SVZ) and subgranular zones. Nevertheless, parenchymal astroglia maintain a latent neurogenic potential that can be reactivated under specific conditions, engaging a program identical to that of niche astroglia. Despite this widespread potential, the regenerative capacity of the mammalian brain is highly reduced compared with non-mammalian vertebrates. The regionalization of the embryonic progenitors into domains of committed progenitors is preserved in adult vertebrates, but while non-mammalian vertebrates continue to generate the same neuron types, in mammals, periventricular domains constituting the V-SVZ converge to generate olfactory bulb interneurons. Cortical and striatal astrocytes also converge toward related neuronal identities, resembling a population of transient developmental neurons. Thus, when astroglia colonized the parenchyma, they carried the niche with them, but their neurogenic potential may have shifted from a reservoir for regeneration to one for plasticity. Paraphrasing Santiago Ramón y Cajal, it is for the science of the future to change, if possible, this harsh evolutionary choice.

## Introduction

Cell-based repair of damaged or diseased brain circuits is one of the major challenges of modern neuroscience. The adult mammalian brain is unable to regenerate, as already noted by Bizzozero in the late 19th century and later crystallized by Ramón y Cajal’s statement that “once development was ended … everything may die, nothing may be regenerated” (Bizzozero, 1893; Ramon y Cajal, 1913; Cajal Santiago Ramon, 1991). The discovery of adult neurogenesis in mammals seemed to challenge this dogma, but in reality, it only scratched its surface, revealing an exception that ultimately confirmed the rule (Altman, 1962; Kaplan, 2001; Nottebohm, 2004). Indeed, adult mammalian progenitors are intrinsically restricted to producing olfactory bulb (OB) interneurons or dentate gyrus (DG) granule cells, a drop in the ocean of neuronal types present in the adult brain.

Yet regeneration is not impossible. Several non-mammalian vertebrates, which share with mammals the same brain bauplan and show deep homologies in neuronal types and circuit organization, retain remarkable regenerative abilities (Alunni and Bally-Cuif, 2016; Joven and Simon, 2018). These differences can only be understood through a comparative lens, by tracing the evolutionary history of neural progenitors. In vertebrates, this history is that of astroglia, a conserved and heterogeneous population of cells. Such an evolutionary framework may shed light on astroglia diversity and inform strategies to modulate their states for neurorepair, whether by enhancing their homeostatic functions or reprogramming their neurogenic potential. In this review, we examine the evolutionary origins of astrocyte neurogenic potential in the context of astroglial development and heterogeneity, how this potential adapted to the mature brain parenchyma, the mechanisms regulating its expression, and the evolutionary shifts in cell-fate competence between non-mammalian and mammalian astroglia.

### Glial cells throughout evolution, the story of all-round multitasking mother cells

**FIGURE 1:**
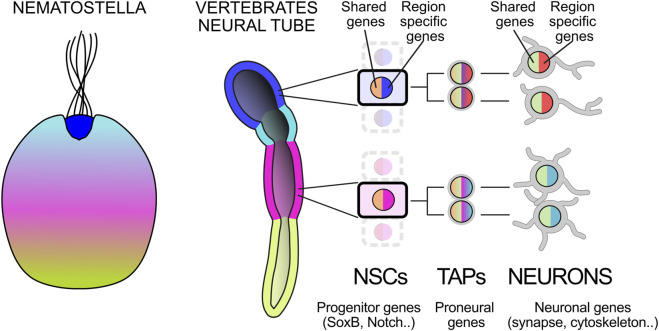
Conserved cellular and molecular mechanisms of neuron genesis and patterning. Throughout evolution, the heterogeneity of neurons and their progenitors has been closely linked to the patterning of the main body axes. Several genes involved in anteroposterior (AP) patterning are conserved between *Nematostella* and the vertebrate neural tube (highlighted in color). Neural stem cells (NSCs) share the expression of genes related to epithelial and progenitor functions (pan-progenitor genes), while differing in some subtype/region-specific genes. These progenitors generate neurons, often through intermediate progenitors that also activatee both genes involved in shared neuronal functions (pan-neuronal genes), together with subtype/region-specific ones. Nematostella is modified from Sachkova et al. (2025); neural tube Puelles et al. (2013).

The generation of neurons is a highly conserved cellular and molecular mechanism across metazoans (Figure 1). Even in distant species such as the cnidarian Nematostella, neural progenitors are induced within an epithelium by BMP inhibition (Bier and De Robertis, 2015) and express SoxB family genes (e.g., Sox2 in vertebrates) and Notch receptors, among others (Hartenstein and Stollewerk, 2015; Kelava et al., 2015). Their daughter cells undergo epithelial to mesenchymal transition, downregulating SoxB and Notch while activating Notch ligands and proneural genes (Rentzsch et al., 2017; 2019). This transition commits them to a neuronal fate, defined by the onset of genes supporting neuronal communication. This program interacts with transcription factors differentially expressed along the body axis, generating region specific neuronal subtypes (Figure 1; Jessell, 2000; Puelles et al., 2013; Sachkova et al., 2025). Interestingly, gene modules for neuronal communication preceded the emergence of neurons, raising the intriguing possibility that their parallel acqusition in different body regions led to the appearance of region-specific neuron types from the very dawn of the neuronal era (Arendt, 2020; 2021; Najle et al., 2023; Sachkova et al., 2025). Neuronal progenitors may likewise have had heterogeneous evolutionary origins, deploying shared regulatory programs alongside spatial patterning mechanisms. Throughout evolution, neurons further diversified in a hierarchy of neuron families, still relying on spatial patterning to generate their variation (Arendt et al., 2019). At the final division, immature neurons inherit from their progenitors a commitment to a region-specific fate through transcriptional codes (Jessell, 2000; Oberst et al., 2019a). Modern neuronal taxonomy approaches, based on transcriptional and electro-morphological features, have shown that, within neuronal families with clear distinct embryonic origin, neurons can further diversify along a seemingly continuous spectrum (Scala et al., 2021). To what extent this further diversification depends on contextual factors rather than intrinsic programs inherited by their progenitors, remains to be established. Notably, positional context can influence morphology and function also independently of transcriptional identity (Hecker et al., 2025; Shainer et al., 2025; Zaremba et al., 2025).

**FIGURE 2:**
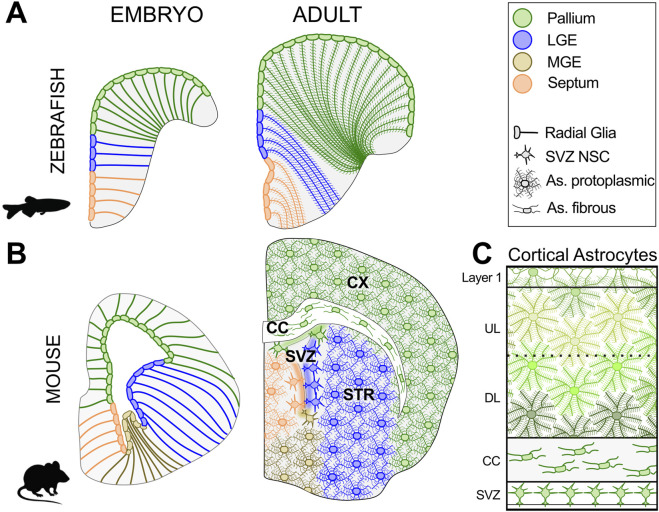
*Astrocyte development and heterogeneity.* Schematic coronal sections illustrate the organization of astroglial cells in the rostral telencephalon of zebrafish and mouse during development (left) and in adulthood (right). Despite the distinct morphogenetic bending of the everted teleost telencephalon, radial glia (RG) are partitioned into conserved dorso-ventral domains (color-coded). At this rostral level, the zebrafish MGE is not visible. In adult zebrafish RG persist, maintaining their regional identity while acquiring mature morphological and functional traits. In adult mice, the embryonic RG scaffold is replaced by astrocytes tiling the parenchyma within the regional boundaries of their RG ancestors. In the cortex, subtypes derived from the same embryonic VZ domain occupy distinct radial niches: neural stem cells (NSCs) in the V-SVZ, fibrous astrocytes in the corpus callosum (CC), protoplasmic astrocytes in deep (DL) and upper (UL) layers with layer-specific features (green shades), and the glia limitans beneath the pia in layer 1. Zebrafish sections adapted from (Chouly and Bally-Cuif, 2024; Morizet et al., 2024); the embryonic mouse section from the Allen Developing Mouse Brain Atlas (E15.5, specimen 100051660, section 248). Abbreviations: UL: upper layers; DL: deeper layers, CC: corpus callosum.

As neuronal specialization increased, so did their reliance on support cells providing a distinct form of “maternal care” ensuring metabolic supply, maintenance of the extracellular milieu, ion and water homeostasis, neurotransmitter clearance, synaptic remodeling, physical compartmentalization from body fluids, and responses to brain lesions (Hartline, 2011; Freeman and Rowitch, 2013; Verkhratsky and Nedergaard, 2018; Sheloukhova and Watanabe, 2024). These support cells are known as glial cells and express specific gene modules that emerged in bilaterians and have remained relatively conserved throughout evolution (Morizet et al., 2024). In invertebrates, glial cells are terminally differentiated cells and do not express neuronal progenitor markers such as the Sox2 homologs (Haim and Rowitch, 2016; Simões and Rhiner, 2017; Egger, 2023; Morizet et al., 2024; Gujar and Wang, 2025). In vertebrates, by contrast, neurogenic and homeostatic functions converged in a single and highly versatile cell type: the astroglia (Freeman and Rowitch, 2013; Morizet et al., 2024). During development, these cells are the first to differentiate from neuroepithelial progenitors maintaining an epithelial organization while extending, on the basal side, a long radial process hence the name radial glia (RG; Rakic, 2003; Mori et al., 2005; Jurisch-Yaksi et al., 2020; Miranda-Negrón and García-Arrarás, 2022). Despite their overall similarity along the neuraxis, RG are regionalized into progenitor domains producing distinct neuron types (Figure 2). After neurogenesis, in non-mammalian vertebrates, the RG scaffold persists lifelong, serving both neurogenic and homeostatic roles (Ganz and Brand, 2016; Jurisch-Yaksi et al., 2020). In mammals, they transform into astrocytes, a closely related cell type that invades and tiles the parenchyma (Figure 2). While most astrocytes specialize in neuron–glia interactions, a restricted subset is retained as neural stem cells (NSCs) in two canonical niches, the ventricular-subventricular (V-SVZ) and subgranular zones (SGZ) (Kriegstein and Alvarez-Buylla, 2009; Bayraktar et al., 2014). This led to the hypothesis that in mammals homeostatic and neurogenic functions became separated again into two sister cell types (Morizet et al., 2024). However, we and others demonstrated that at least in some brain regions, parenchymal astrocytes retain a latent neurogenic potential, and in specific conditions can generate neurons outside canonical neurogenic niches (Magnusson et al., 2014; Nato et al., 2015; Péron and Berninger, 2015). This indicates that the similarities between astrocytes and non-mammalian RG cells are wider than previously thought and the adult brain parenchyma can be permissive for neuronal progenitor maintenance and activity.

## Embryonic neurogenesis

During embryonic development, the RG forms a pseudostratified epithelium termed ventricular zone (VZ) and represents the main neuronal progenitors (Noctor et al., 2001; Malatesta et al., 2003; Anthony et al., 2004). These cells have long been recognized as glial cells for morphological and molecular characteristics (Ramón y Cajal, 1909; Borrett et al., 2020); (Figure 2). Morphogen gradients subdivide the VZ into domains of committed progenitors generating specific neuron subsets through characteristic and highly conserved transcription factor codes (Jessell, 2000; Puelles et al., 2000; Reichert and Simeone, 2001; O’Leary et al., 2007; Appan et al., 2023; Sachkova et al., 2025).

RG progenitor activity progresses through three highly stereotyped sequential phases: amplification, neurogenesis, and gliogenesis, whose timing is asynchronous across the RG population (Lin et al., 2021). After early symmetric expansion, RG cells switch to asymmetric divisions, producing either neurons directly or *via* transit-amplifying progenitors (TAPs) that divide rapidly but are more fate-restricted and expand underneath the VZ, in a specific layer called the subventricular zone (SVZ; Angevine et al., 1970; Noctor et al., 2004; 2007). Neurogenic production by RG cells follows a highly deterministic program, characterized by a relatively fixed number of divisions and mostly resulting in strikingly stable clonal spatial organization (Gao et al., 2014; Lin et al., 2021). According to the theory of radial units, this localized pattern of activity preserves early spatial patterning (Rakic, 1988; Noctor et al., 2001).

In mammals, the strong radial thickening and tangential expansion of the brain wall induced the expansion of the SVZ, that started to be populated also by ventricle-detached RG cells, the outer RG (Florio and Huttner, 2014; Pilz et al., 2013; Lui et al., 2011; Kawaguchi, 2020). In gyrencephalic species, outer RG cells are particularly abundant and form a distinct layer. Thus, a key evolutionary innovation in mammalian RG cells is their capacity to generate neurons outside the ancestral peri-ventricular epithelial niche.

## Astrocyte development and heterogeneity

In vertebrates, astrocytes are highly versatile and conserved cells that sustain neurogenic and homeostatic roles, but also modulate synaptic plasticity and information processing. They respond to neurotransmitters and directly communicate with neurons through gliotransmission, maintaining extensive intercellular coupling *via* gap junctions (Balmaceda-Aguilera et al., 2012; Durkee and Araque, 2019; Mu et al., 2019; Jurisch-Yaksi et al., 2020; Cooper et al., 2025).

Whether astrocytes form a homogeneous population of multifunctional cells or instead comprise specialized subtypes, either intrinsically determined or dynamically shaped by contextual factors, remains an open and actively investigated question (Bayraktar et al., 2014; Hennes et al., 2025; Kwon et al., 2025). This diversity is rooted in the developmental and evolutionary history of RG, and reconstructing this history is therefore a critical step to decipher it (Figure 2).

### Astrocytes in non-mammals

In non-mammalian vertebrates RG persists after development, but progressively reduces proliferation, transforming the VZ from a pseudostratified to a single-layered epithelium. As neurons accumulate and mature, RG develop fine processes resembling astrocytic leaflets and increase neuron contacts (Figure 2A; Jurisch-Yaksi et al., 2020). This morphological maturation is accompanied by a progressive shift from neurogenic to homeostatic functions (Diaz Verdugo et al., 2019; Mu et al., 2019; Raj et al., 2020; Morizet et al., 2024). In zebrafish, transcriptional differences between RG in different domains are more pronounced during embryonic stages, possibly reflecting the active role of patterning factors and selector genes in defining regional identity. These differences are gradually attenuated at the end of development, while RG converges toward a shared functional state, with only residual regional specificities (Raj et al., 2020; Mitic et al., 2024; Morizet et al., 2024). Of note, in adults, regional transcriptional differences are largely lost upon neurogenic activation, despite the generation of distinct neuronal types (Lange et al., 2020), implying that non-transcriptional mechanisms, such as chromatin modifications, may preserve positional information in adult astroglia.

In birds, RG persistence is accompanied by the emergence of parenchymal astrocytes, morphologically and transcriptionally related cells that detach from the ventricle and migrate into the parenchyma (Falcone, 2022; Ciani et al., 2024; Morizet et al., 2024). These cells are rare in reptiles and likely evolved independently in birds and mammals as an adaptation to increased brain complexity, though their functions in birds remain largely unexplored.

### Astrocytes in mammals

In mammals, during development RG progressively acquire astrocytic features such as increased branching and vascular contacts, paralleling neuronal maturation (Schmechel and Rakic, 1979a; 1979b; Takahashi et al., 1990; Mori et al., 2005). Around mid-gestation, a gliogenic switch generates a first wave of astroblasts migrating into the parenchyma, followed by a second wave at the end of neurogenesis (around P0–P3 in mice), when residual RG retract their processes, detach from the ventricle, and transform into astrocytes (Gressens et al., 1992; Ge et al., 2012; Clavreul et al., 2022), while the ventricular layer becomes lined by ependymal cells (Bayraktar et al., 2014; Redmond et al., 2019). In the mouse neocortex only about 20% of the EMX1+ RG generate astrocytes, while the others are consumed in neurogenic differentiation (Gao et al., 2014; Lin et al., 2021; Shen et al., 2021). In parallel, by late stages of neurogenesis a population of Olig2+ progenitors, called the Tri-IPCs, contributes oligodendrocytes, olfactory bulb-like interneurons and astrocytes in the cortex (Zhang et al., 2020; Wang et al., 2025). Following RG scaffold disassembly, immature astrocytes proliferate locally and tile the parenchyma in a near-uniform lattice (Ge et al., 2012; Clavreul et al., 2019; Endo et al., 2022). Clonal analyses show stochastic dispersion, which however is constrained within their domain of origin (Hochstim et al., 2008; Tsai et al., 2012); (Figure 2). Neither age, injury, nor the loss of neighboring astrocytic populations causes astrocytes to cross these boundaries (Tsai et al., 2012), suggesting that their regional identity may be intrinsically determined. The overall subdivision of these domains is highly conserved in vertebrates (Puelles and Medina, 2002; Puelles et al., 2013; Luzzati, 2015), suggesting conservation and/or co-evolution of regional astrocytes-neurons interaction (Figure 2). As in zebrafish, mammalian astrocytes converge towards similar gene expression profiles, consisting in a strong core of shared gene modules, with subtler region-specific adaptations (Morel et al., 2017; Lozzi et al., 2020; Endo et al., 2022; Kwon et al., 2025; Morel et al., 2017; Lozzi et al., 2020; Endo et al., 2022). Notably, similarities are higher within major divisions of the brain, such as pallium *versus* subpallium, while sharp transcriptional discontinuities separate them, a pattern that mirrors neuronal regionalization and may reflect the evolutionary history of these domains (Endo et al., 2022).

#### Astrocyte intra-regional diversity

Beyond this tangential organization, mammalian astrocytes exhibit radial, intra-regional heterogeneity in morphology and transcriptional profile (Figure 2C). Classical types include protoplasmic, fibrous, and glia limitans astrocytes, respectively occupying gray matter, white matter, and subpial layers (Verkhratsky and Nedergaard, 2018). Additional region-specific forms, such as Bergmann glia of the cerebellum or NSCs of the V-SVZ and SGZ, display specialized morphologies and molecular signatures (Bayraktar et al., 2014; O’Shea et al., 2024; Bocchi et al., 2025; Cerrato et al., 2025; Hennes et al., 2025). Some of these types derive from at least partially distinct lineages (García-Marqués and López-Mascaraque, 2013; Cerrato et al., 2018; 2025; Bocchi et al., 2025) and retain their fate after transplantation (Kantzer et al., 2021). In the cortex, protoplasmic astrocytes originate from early migrating glioblasts that proliferate extensively postnatally, while fibrous astrocytes derive from later detaching RG that show limited proliferation (Ge et al., 2012; Clavreul et al., 2019; Bocchi et al., 2025). Neurogenic astrocytes of the V-SVZ also constitute a distinct astrocytic lineage derived from almost all telencephalic progenitor domains (Figure 2B) (Merkle et al., 2007; Young et al., 2007). They diverge from the lineage of their neuronal siblings during the early gliogenic wave, around E15.5 in the mouse (Fuentealba et al., 2015). These cells maintain a radial process anchored to the pial surface until the end of development (Merkle et al., 2007) and differentiate into astrocytes with only minimal expansion, probably because of the limited volume of their niche (Fuentealba et al., 2015; Furutachi et al., 2015). By contrast, the SGZ neurogenic astrocytes originate from a single progenitor domain and secondarily expand into the SGZ, displaying an intermediate behavior between glioblasts and outer RG cells (Berg et al., 2019; Caramello et al., 2021). These data reveal the presence of separate radial niches for astrogliogenesis within the same region, at least partially associated with distinct astrocyte types and developmental patterns.

Further variability exists within these astroglial niches such as the layer morphological features of cortical protoplasmic astrocytes (Figure 2C; Bayraktar et al., 2020). The stochastic dispersion of these astrocytes during development (Clavreul et al., 2019) and their adaptation to changes in neuron positioning (Bayraktar et al., 2020) suggests that layer specific properties are regulated by local cues. In parallel to these regional specializations, single cell RNA-seq have also identified astrocyte subtypes shared between different regions, possibly linked to specializations of their core homeostatic functions (Endo et al., 2022).

In general, understanding the intrinsic constraints of astrocyte identity is hampered by the dynamic changes that environmental and cellular interactions, including neuronal activity, can normally induce in astrocyte states (Kwon et al., 2025). An extreme example of such plasticity is astrocyte responses to injury. In these conditions, they become reactive, undergoing morphological, molecular, and functional changes (Sofroniew, 2009; Escartin et al., 2021; Clayton and Liddelow, 2025). Reactive astrocytes can transiently downregulate core homeostatic genes and acquire distinct non-overlapping states, characterized by further variability. After invasive injuries they can reactivate proliferation, upregulate developmental programs and re-differentiate into barrier-forming types, showing similarities with the glia limitans (O’Shea et al., 2024).

In conclusion, the astroglial lineage across vertebrates shows a temporally regulated transition between neurogenic and homeostatic functions. While in the adult these cells maintain a strong regional segregation, they converge on a similar gene expression profile, with minor inter- and intra-regional differences. Understanding the evolutionary history of the differentially activated gene modules and their co-evolution with neurons, will provide a broader framework to understand and classify astrocyte subtypes and states.

Nonetheless, astroglial cells are characterized by a highly plastic capacity, suggesting they may represent largely multifunctional cells. In mammals, the tiling of the parenchyma allowed for higher heterogeneity within the population (Figure 2C), adapting to more local and specialized interactions with neurons and other brain cells.

## Adult neurogenesis in non-mammalian vertebrates

**FIGURE 3:**
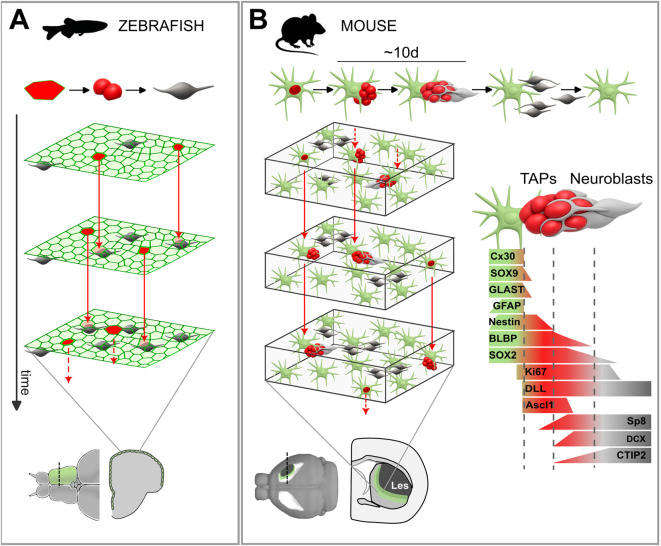
*Dynamics of neurogenic activation in the fish pallium and mouse striatum*. **(A)** The upper row illustrates the lineage progression from active radial glia (RG) to transit-amplifying progenitors (TAPs) and neuroblasts. Below, schematic views of the apical surface of the zebrafish pallial ventricular zone (VZ) at three successive time points are shown. Neurogenic activation is depicted in red, and red arrows indicate its outcome in the subsequent frame. **(B)** Same as in **(A)**, but for the lesioned (Les) mouse striatum. A red nucleus marks proliferating cells, and red arrows connect dividing cells across time points. On the right, the main molecular markers associated with lineage progression are indicated according to (Luzzati et al., 2011; Magnusson et al., 2014; Nato et al., 2015; 2025; Fogli et al., 2024).

Neurogenic hotspots in adult RG have been described throughout the neuraxis across all non-mammalian vertebrate groups (Alvarez-Buylla et al., 1990; Chapouton et al., 2007; Hinsch and Zupanc, 2007; Kaslin et al., 2008; Berg et al., 2010; Vellema et al., 2010; Bonfanti et al., 2011; Alunni and Bally-Cuif, 2016; Ganz and Brand, 2016). The best characterized of these niches is the zebrafish dorsal pallium, where RG progenitors form a monolayer with their apical side facing the brain surface (Figures 2A, 3A). This peculiar organization of the everted telencephalon allows intravital imaging and global analysis of RG spatio-temporal dynamics (Barbosa et al., 2015; Dray et al., 2015; 2021a; 2021b). Unlike development, adult RG are mostly quiescent: only about 4% undergo activation each day (Than-Trong et al., 2020). Daughter cells undergo direct differentiation or a minimal intermediate amplification (one to two divisions; Barbosa et al., 2015; Furlan et al., 2017; Than-Trong et al., 2020). New pallial neurons are continuously added through three hierarchically-organized and intermingled NSC populations characterized by increasing activation rates: a “source pool” that enables growth of the NSC population, a “reservoir pool” undergoing invariant asymmetric self-renewal while giving rise to an “operational pool” displaying neurogenic activity (Barbosa et al., 2015; Furlan et al., 2017; Than-Trong et al., 2020). The operational pool divides mainly asymmetrically but is characterized by unbalanced stochastic fate choice, in which the probability of terminal differentiation increases with time. Thus, NSC maintenance relies on both invariant asymmetric division and population asymmetry.

Spatial statistics revealed that NSC activation events are randomly distributed in space (Dray et al., 2021a). Nonetheless, local feedback inhibition exerted by intermediate neuronal progenitors, involving Notch signaling, biases the localisation of new NSC activation events farther away. Overall, these spatiotemporal coordination dynamics generate an “intrinsic niche” contributing to NSC population homeostasis in physiologic conditions (Figure 3A; Dray et al., 2021a; 2021b). Interestingly, RG clones expand proportionally to hemispheric growth maintaining stable size and position, similar to developmental radial units (Than-Trong et al., 2020). This contrasts with neutral drift dynamics in epithelia such as epidermis or testis, where stochastic fate leads to clonal competition, with the progeny of individual stem cells progressively expanding at the expense of neighboring clones, which conversely shrink (Klein and Simons, 2011; Simons and Clevers, 2011). The cellular and molecular mechanisms that maintain this spatial stability are still unknown.

RG proliferation can strongly increase after brain lesions, recruiting also normally inactive populations, such as in the retina or the spinal cord, leading to efficient regeneration (Kyritsis et al., 2012; Alunni and Bally-Cuif, 2016; Foley et al., 2024; Morizet et al., 2025). Divisions become largely symmetric to boost neuron output, while other RG cells enter deep quiescence, balancing maintenance and repair (Barbosa et al., 2015). Thus, zebrafish neurogenesis is a slow but constitutive, developmental-like process, yet highly context-dependent, allowing regeneration after lesions.

## Adult neurogenesis in mammalian canonical niches

In mammals, a subset of astrocytes continues to generate neurons, and to a lesser extent glial cells, in two specific niches: the V-SVZ and the SGZ of the DG (Altman, 1962; Doetsch et al., 1999; Seri et al., 2001; 2004; Gage, 2002; Alvarez-Buylla and Lim, 2004; Bonzano et al., 2018; for reviews see: Lim and Alvarez-buylla, 2014; Gonçalves et al., 2016; Llorens-Bobadilla and Martin-Villalba, 2017; Obernier and Alvarez-Buylla, 2019; Vicidomini et al., 2019). These NSCs retain RG traits such as defined location, a planar sheet-like organization, apico-basal polarity, some contact the ventricles in the V-SVZ, and have a radial process contacting neurons in the SGZ. Once activated, NSCs generate TAPs, which divide from 1–2 times in the SGZ to 3–4 times in the V-SVZ, before differentiating into neuroblasts. In both niches, NSCs also have a restricted cell fate potential, generating specific subsets of olfactory bulb interneurons in the V-SVZ and DG granule cells in the SGZ. In the DG, the new cells are added to pre-existing neurons while in the OB they are turned over (Imayoshi et al., 2008).

### Spatio-temporal dynamics and self-renewal

In mammals, neurogenesis is highly active during early life, reflecting a key conserved role in postnatal brain development. In adulthood, it persists at much lower levels and varies substantially across species, possibly contributing to long-term circuit plasticity in specific contexts (Amrein et al., 2011; Barker et al., 2011; Bonfanti et al., 2011; Kempermann, 2012; Sailor et al., 2017; Charvet and Finlay, 2018; Kempermann et al., 2018; Sorrells et al., 2021). Whether this decline reflects NSC exhaustion, deep quiescence or an unfavorable niche environment has been the subject of intense debate (Kalamakis et al., 2019; Harris et al., 2021; Carvajal Ibañez et al., 2023). The NSCs in the V-SVZ and SGZ are mostly quiescent, and only 0.15%–0.3% of them undergo activation each day, ∼30-fold less than adult zebrafish NSCs (Calzolari et al., 2015; Ziebell et al., 2018). Clonal analyses and intravital imaging in the V-SVZ and SGZ suggested that most NSCs undergo a few rounds of divisions, generating multiple waves of fast-expanding progeny before exhaustion (Calzolari et al., 2015; Pilz et al., 2018) or terminal astrocyte differentiation (Encinas et al., 2011). In parallel to this “disposable NSC population”, other studies in the SGZ identified cells with longer self-renewal capacities (Bonaguidi et al., 2011; Bottes et al., 2021), possibly acting as the zebrafish “reservoir pool” (Dray et al., 2021b). Alternatively, NSC behavior may distribute over a fluid spectrum of self-renewal potential. As clonal analyses possess intrinsic limitations in the discrimination of self-renewal strategies (Parigini and Greulich, 2020), more extensive and protracted observations, akin to those done in the zebrafish would be required to resolve this issue.

In the V-SVZ, a combination of symmetric self-renewing divisions (20%) and symmetric consuming divisions (80%) gradually reduces the population of NSCs contacting the ventricle, also known as Type B1 cells (Obernier et al., 2018). This reduction however, is counterbalanced by the increase of basal NSCs or Type B2 cells, originally thought to represent non-neurogenic SVZ astrocytes but recently shown to also have neurogenic potential (Cebrian-Silla et al., 2025). A comprehensive model of mouse V-SVZ NSC dynamics has been proposed by Basak et al. based on long-term lineage tracing, clonal analyses, and mathematical modeling (Basak et al., 2018). Similar to classic stem cell systems, NSCs divide symmetrically and the daughter cells stochastically choose between returning to quiescence (maintenance) or progressing into differentiation (exhaustion). The probability of taking either fate correlates with the amount of neighboring NSCs, so that reducing numbers sustains maintenance while crowding stimulates exhaustion (Basak et al., 2018). In line with this model, the reduced NSC density observed in the aged niche may increase the probability of self-renewing asymmetric divisions in which one of the daughter cells return to quiescence (Bast et al., 2018; Harris et al., 2021; Wu et al., 2023; Cebrian-Silla et al., 2025), further contributing to the lifelong maintenance of neurogenesis. As for the zebrafish pallium the number and relative position of NSCs in long term clones is remarkably stable and does not show any neutral drift (Basak et al., 2018). The V-SVZ may thus be viewed as a mosaic of “restricted niches”, maintained by local factors, whose nature however remains to be established. This model may also apply to other embryonic and adult niches in vertebrates, supporting the organization in “radial units”.

Overall, these findings reveal remarkable conservation in NSC spatio-temporal dynamics between non-mammalian and mammalian neurogenic astroglial progenitors.

## Astrocyte identity of neural stem cells

Lineage-tracing and ablation studies, together with molecular, ultrastructural, and electrophysiological analyses, have clearly demonstrated the astroglial nature of mammalian NSCs (Doetsch et al., 1999; Seri et al., 2001; Fukuda et al., 2003; Imura et al., 2003; Liu et al., 2006). These cells possess end-feets on blood vessels, participate in tripartite synapses and respond to neuronal activity (Doetsch et al., 1997; Song et al., 2012; Paez-Gonzalez et al., 2014; Young et al., 2014; Moss et al., 2016; Yeh et al., 2018).

Single-cell RNA-seq analyses in adult niches further revealed that, as for other stem cell populations, adult NSCs span along a continuum of activation states (Llorens-Bobadilla et al., 2015; Shin et al., 2015; van Velthoven and Rando, 2019; Belenguer et al., 2021). Neurogenic activation typically involves an initial transition from a deeply quiescent to a primed state, which is not yet active but already displays transcriptional and metabolic changes that shift cells toward neuronal programs and make them more prone to progress to full neurogenic activation. Interestingly, in fish, core homeostatic modules of astrocyte function are upregulated during quiescence and downregulated in the active state (Morizet et al., 2024). Accordingly, quiescent RG showed the strongest similarities with mammalian astrocytes (Morizet et al., 2024; 2025). Similar results were obtained in the mouse V-SVZ (Codega et al., 2014; Llorens-Bobadilla et al., 2015; Dulken et al., 2017; Kalamakis et al., 2019; Kremer et al., 2024) and SGZ (Shin et al., 2015; Harris et al., 2021), where it was further shown that the active cells resemble embryonic RG progenitors (Borrett et al., 2020). This suggests that while neurogenic and homeostatic functions coexist at the population level, they represent at least partially alternative states at the single-cell level. Whether embryonic RG cells, whose astrocytic traits increase during development, undergo a similar functional switch remains to be determined. Notably, during mid-gestation some RG enter quiescence, and this has been associated with an astroglial transition (Schmechel and Rakic, 1979b; Fuentealba et al., 2015; Furutachi et al., 2015; Bocchi et al., 2025). Cyclin-dependent kinase inhibitors have also been proposed to play a role in this switch (Lee et al., 2024).

The antagonism between homeostatic and progenitor states further supports the idea that vertebrate astroglia arose from the fusion of two distinct ancestral cell types (Morizet et al., 2024). These dual states are likely still governed by partially independent gene regulatory networks, and accordingly, in the V-SVZ their interconversion requires epigenetic remodelling, which is particularly evident in the methylome of primed NSCs as compared with dormant ones (Kremer et al., 2024). The dormant NSC methylome is very similar to that of striatal astrocytes as for instance they displayed low-methylation regions (LMR) near to genes regulating astrocyte homeostatic functions. By contrast the primed NSC methylome resembled the one of active NSCs as they showed LMR at genes involved in neuronal differentiation.

If this duality is so deeply rooted in astroglial cells, a natural question arises as to whether it extends to parenchymal astrocytes.

## Parenchymal astrocyte neurogenic potential

Parenchymal astrocytes express SOX2, a highly conserved neuronal progenitor marker but are normally quiescent and long considered terminally differentiated cells (Arvidsson et al., 2002; Dimou and Götz, 2014). However, after cortical lesion, local reactive astrocytes display neurogenic potential *ex vivo* in both mice and humans (Buffo et al., 2008; Sirko et al., 2013; 2023). Whether this potential could be expressed *in vivo* or merely reflected an *in vitro* artifact initially remained unclear. Outside the canonical niches, the brain parenchyma was long considered strictly gliogenic and non-permissive for neurogenesis, a view supported by the glial fate of NSCs transplanted into these regions (Herrera et al., 1999; Lim et al., 2000; Shihabuddin et al., 2000; Eriksson et al., 2003). However, concurrent and subsequent studies revealed that, under specific conditions, the parenchyma can be permissive for neuronal genesis.

**FIGURE 4:**
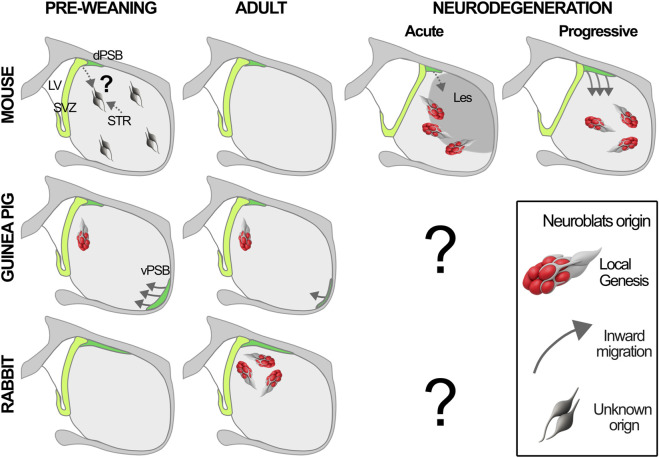
*A comparative framework of striatal neurogenesis in mammals*. Schematic coronal brain sections at the level of the caudate-putamen (Cpu) showing the presence and origin of newborn neurons in different conditions (pre-weaning, adult physiology or after neurodegeneration) across three mammalian species. The origin of immature LGE-class neuroblasts observed in mice during the pre-weaning period remains to be established (Nato et al., 2025). After acute lesion (Les) inward migration has been observed after stroke (Liu et al., 2009) but not QA (Nato et al., 2015; Fogli et al., 2024). The distribution of the neurogenic foci is based on the QA model. Guinea pig data are from (Luzzati et al., 2014), rabbit data from (Luzzati et al., 2006) and unpublished observations. dPSB and vPSB = dorsal and ventral Pallial Subpallial Boundary.

In contrast to the minimal neurogenesis seen in mice and rats (Dayer, 2005), the rabbit caudate nucleus contains numerous neuroblasts, partly organized in long chains, constitutively generated throughout life (Luzzati et al., 2006). Intraventricular injections of the cell tracer CTG and 3D reconstructions demonstrated that these cells are not V-SVZ derived. Instead, the presence of local progenitors was confirmed by BrdU+ neuroblasts migrating out of striatal explants taken from animals treated with BrdU 2 h before sacrifice. The intra-striatal neurogenic activity was identified in clusters of actively dividing Ki67+ cells, associated with the neuroblast chains, closely resembling the clusters of TAPs in canonical neurogenic niches. These TAPs-like cells also expressed the glial marker BLBP (Anthony et al., 2004), suggesting a local astrocyte origin, although this could not be directly verified. This provided the first evidence that mature brain parenchyma can host neuronal progenitor activity. Similar V-SVZ-independent neurogenic foci were subsequently found in the distal end of the external capsule associated with the ventral Pallial Subpallial Boundary (vPSB) of the guinea pig around weaning (Luzzati et al., 2014) and in the striatal parenchyma of a model of progressive degeneration in mice (Luzzati et al., 2011), indicating that local neurogenesis can also occur in rodents (Figure 4). The striatal TAPs-like cells expressed typical markers of V-SVZ TAPS, as BLBP, SOX2, SOX9, EGF receptor, and pan-Dlx, indicating a GABAergic fate (Figure 3B; Doetsch et al., 2002; Cheng et al., 2009; Sun et al., 2017).

The direct demonstration that these parenchymal neurogenic foci originate from local astrocytes came from two independent studies in stroke (Magnusson et al., 2014) and quinolinic acid (QA; Nato et al., 2015) lesion models. In both, the foci and their neuronal progeny could be lineage traced from Cx30+ or Glast+ astrocytes, but not NG2+ oligodendrocyte progenitors, and by intrastriatal viral injection of a Lenti-GFP or Ad:GFAP-Cre virus before the lesion. As in canonical niches, the neurogenic activation of striatal astrocytes required Notch downregulation (Magnusson et al., 2014), and was associated with Nestin expression and neurospherogenic potential (Nato et al., 2015). Interestingly, unlike other lesioning paradigms, including stroke, the QA lesion did not elicit almost any neuroblast migration from the V-SVZ (Figure 4; Nato et al., 2015). These studies however, did not resolve the organization of the parenchymal niche including the prevalence, spatial distribution and dynamics of these ectopic astroglial NSCs.

### Spatio-temporal dynamics of astrocyte neurogenic activation in the striatal niche

The main hallmarks of neurogenic niches, such as anatomical confinement, planarity and apico-basal polarity are largely lost in the 3D lattice of parenchymal astrocytes, raising the question of how their neurogenic potential may have adapted to this new environment. In the QA model, clonal analyses showed that neurogenic foci originate from the local expansion of individual astrocytes (Fogli et al., 2024). These foci persist for about 10 days, during which they grow and mature while continuously producing post-mitotic neuroblasts (Figure 3B). As each TAP clone completes its cycle, a new one emerges, maintaining a steady-state turnover. Astrocytes undergo only a brief activation that seeds neurogenic clones, after which in most cases they re-enter quiescence maintaining close contact with their daughter cells. According to the model of symmetric division and competition for a restricted niche (Basak et al., 2018), this high maintenance rate may result from lower astrocyte density in the striatum, increasing the likelihood of niche re-rentry.

By using the neurogenic foci as a proxy for the location of astrocyte activation events over the last 10 days, we demonstrated that these events are randomly distributed, with only a minor inhibitory effect from nearby clusters, closely matching the behaviour of zebrafish pallial RGs (Dray et al., 2021a). The activation rate was remarkably similar in the V-SVZ and in the striatal neurogenic area, at about 0.2%–0.4% astrocytes/day (Calzolari et al., 2015; Fogli et al., 2024), suggesting a similar prevalence of neurogenic progenitors in these regions, thus potentially encompassing the entire striatal astrocyte population.

Thus, despite the different tissue organization, when activated, parenchymal neurogenesis unfolds through the same cellular mechanisms and spatio-temporal dynamics as in planar neurogenic niches across evolution.

### TAP contribution to neurogenic outcome

Mammalian neurogenic production relies more heavily than other vertebrates on TAPs, particularly in late developmental phases and in adult life. This may compensate for the need to boost both neuronal output and homeostatic support, despite their intrinsic trade-off. While TAPs are a crucial step in regulating the neuronal outcome, their behaviour is poorly studied. During development, TAPs typically follow deterministic division patterns, performing a few symmetric divisions before differentiating (Lin et al., 2021). Adult TAPs were initially thought to retain this rigid logic: in V-SVZ they divide about three times (Ponti et al., 2013; Calzolari et al., 2015). However, intermediate progenitor cell activity can be modulated by different neurogenic stimuli, as clearly shown in the DG (Kronenberg et al., 2003). More recently, clonal analyses (Bast et al., 2018) and live imaging (Pilz et al., 2018) revealed that most V-SVZ and SGZ TAPs divide symmetrically, while ∼25% divide asymmetrically, displaying highly variable division patterns. Although TAP expansion is lower in the SGZ, and closer to zebrafish dynamics, these results imply that TAP behavior is less deterministic than previously assumed (Götz, 2018). In the QA lesion, the TAPs clones had highly variable size and cellular composition. Mathematical modelling revealed that this heterogeneity is driven by a stochastic division process acting in parallel to an accelerating differentiation propensity, that introduces a deterministic factor constraining their expansion and ultimately leading to their exhaustion (Fogli et al., 2024). The high heterogeneity among striatal neurogenic foci may thus underlie a locally regulated control of neuronal output, and exploring the underlying cellular and molecular mechanisms could provide valuable insights into how parenchymal neurogenesis is modulated.

### Regulatory mechanisms of NSC activation and maintenance

Overall, at least in the striatum and likely also in the neocortex, the main difference between canonical and parenchymal neurogenic niches lies not in the presence of quiescent NSCs, but in how their activity is regulated. Canonical niches provide protected and permissive environments that preserve progenitor stemness, maintain a quiescent pool for lifelong neurogenesis, and coordinate NSC activation by integrating multiple physiological and pathological cues. These mechanisms have been extensively reviewed elsewhere (Fuentealba et al., 2012; Lim and Alvarez-Buylla, 2014; Vicidomini et al., 2019; Llorente et al., 2022; Chaker et al., 2024; Foley et al., 2024). In adult canonical niches NSCs are mostly quiescent, but their activity can be stimulated by several external cues (Kempermann et al., 2015; Song et al., 2016; Obernier and Alvarez-Buylla, 2019; Caron et al., 2022; Chaker et al., 2024). Experience-dependent cues, such as physical exercise, environmental enrichment or learning can increase SGZ neurogenesis by multiple pathways including the release of BDNF, modulation of local GABAergic tone, and excitatory inputs onto NSCs (van Praag et al., 2000; 2002; Catavero et al., 2018; Liu and Nusslock, 2018; Li et al., 2023). Some stimuli can elicit localized effects such as nutritional state or pregnancy that activate specific V-SVZ domains to generate specific OB subtypes (Paul et al., 2017; Chaker et al., 2023). By contrast, in all known vertebrate niches examined so far, brain lesions induce a strong activation of both quiescent and primed NSCs (Arvidsson et al., 2002; Kernie and Parent, 2010; Jurisch-Yaksi et al., 2020; Morizet et al., 2025). In mammals, these responses are partly mediated by inflammatory cytokines (IL-6, CNTF, TNFα, Interferons; Llorens-Bobadilla et al., 2015; Belenguer et al., 2021).

These regulatory mechanisms are mediated by multiple cellular components, including blood vessels, microglia, neuronal afferents, and astrocytes, all of which are also present throughout the parenchyma. In non-mammalian vertebrates, RG cells engage with these elements directly within the parenchyma, and this environment likely represents an integral component of their niche (Jurisch-Yaksi et al., 2020). In mammals, the transformation of RG into astrocytes has profoundly modified the anatomical relationship of individual cells, further parcellating these ancestral niches. These new microenvironments, however, still regulate the delicate balance between quiescence and activation of astroglial stem cell potential. Accordingly, key pathways involved in NSC quiescence and maintenance, including Notch (Imayoshi et al., 2008; Engler et al., 2017; Lampada and Taylor, 2023; Morizet et al., 2025), BMP (Lim et al., 2000), β1-integrin (Porcheri et al., 2014), S1P (Codega et al., 2014), and GABA (Liu et al., 2005; Song et al., 2012; 2016) are strongly active in parenchymal astrocytes (Lim et al., 2000; Robel et al., 2011; Magnusson et al., 2014; Nagai et al., 2019; Singh et al., 2022). In line with the developmental switch between neurogenic and homeostatic states, these pathways also regulate astrocyte morphogenesis (Meyers and Kessler, 2017; Cheng et al., 2023; Guo et al., 2023; Gonzalez and Reinberg, 2025). The adult parenchyma can thus be considered as a quiescent neurogenic niche, subdivided into a mosaic of multiple sub-niches.

Unlike in canonical niches, abrogation of these pathways is not sufficient to activate neuronal production from parenchymal astrocytes (Lim et al., 2000; Porcheri et al., 2014). However the underlying changes in astrocyte states have been investigated in detail only for the Notch signalling (Magnusson et al., 2020; Zamboni et al., 2020). Notch pathway is a highly conserved regulator of NSC activity whose abrogation induces neurogenic activation across metazoans, from cnidarians (Richards and Rentzsch, 2015) to vertebrates (Rothenaigner et al., 2011; Shimojo et al., 2008; Imayoshi et al., 2010). Deletion of Rbpj, a central Notch effector, drives striatal and cortical astrocytes into a shallow quiescent or “primed” state, which occasionally progresses to overt neurogenesis in the medial striatum and medial cortex (Magnusson et al., 2014; 2020; Zamboni et al., 2020). Brain lesions can independently induce similar primed states in cortical and striatal astrocytes (Zamboni et al., 2020; Kremer et al., 2024) and at least in the striatum it involves methylome changes that closely resemble those observed during V-SVZ astrocyte activation. These observations suggest that, as in canonical niches, parenchymal astrocytes may transit to a primed state and only subsequently to actual neurogenic state. However, in some conditions, such as stab injury or after Notch abrogation, this primed state does not progress into neurogenesis (Buffo et al., 2008; Magnusson et al., 2020; Zamboni et al., 2020), suggesting that reawakening of neurogenic competence and its expression may be regulated by distinct factors. The molecular factors controlling these states in physiologic and pathologic conditions remain to be established.

Throughout evolution, brain lesions not only strongly stimulate NSCs activation, but also induce profound changes in homeostatic functions of glial cells activating context- and time-dependent states, also called reactive states, that limit damage and support repair (Götz et al., 2015; Sofroniew, 2020; Escartin et al., 2021; Clayton and Liddelow, 2025). Given the dual nature of vertebrate astroglia, and the further elaboration of astrocyte functions and heterogeneity in mammals, a non-trivial and still mostly unresolved question is how these multiple functions and states are regulated and if they can coexist only at the population or also at the individual cell level. In both rodents and humans, the reawakening of astrocyte neurogenic competence, measured with the neurosphere assay, was found associated only with invasive injuries, where the type of astrocytes reactivity include cells that proliferate and transiently re-acquire more immature features (Buffo et al., 2008; Sirko et al., 2013; 2023). In both stroke and QA model, further neurogenic activation of primed astrocytes is typically delayed relative to the peak of reactivity, possibly associated with a more reparative phase, and at least in the QA was independent from early proliferation or expression of C3, a marker of a reactive astrocyte subtype (Fogli et al., 2024). This indicates that the neurogenic program is compatible with multiple reactive sub-states, within the context of the sub-acute phase of an invasive injury.

The tight temporal control of the striatal neurogenic response is paralleled by an equally tight spatial control. Indeed, although the neurogenic potential is widespread in the striatal parenchyma, its activation is typically focal (Figure 4). Following QA-induced lesions, the neurogenic region aligns with the lesion border, mostly to its rostral and ventro-medial part (Fogli et al., 2024). In contrast, in mouse models of progressive degeneration, neurogenic activation is shifted toward more lateral striatal domains (Luzzati et al., 2011), remains confined dorso-medially in normal rabbits (Luzzati et al., 2006), and ventro-laterally in guinea pigs at weaning (Luzzati et al., 2014). Such localized activation generates neurons interacting with specific neuronal networks, raising the question of whether these same circuits may contribute to neurogenic activation. In canonical niches NSCs can be directly modulated by neurotransmitters and are highly sensitive to neural activity (Song et al., 2012; Paez-Gonzalez et al., 2014; Káradóttir and Kuo, 2018). The evolutionary transformation of RG into astrocytes resulted in more localized interactions with specific neuronal populations, allowing greater flexibility in neuron–astroglial networks. While specializing in local circuit-specific interaction, mammalian astrocytes may have thus also refined the circuit-specific regulation of their neurogenic capacity.

Altogether, these findings indicate that similarly to neurogenic niches, parenchymal neurogenic activation may pass through multiple sequential steps, each potentially responding to specific signals. While the spatial organization of different neurogenic niches and their activatory stimuli remains to be established, the stability of astrocyte units provide a framework for highly tunable spatio-temporal dynamics of adult neurogenesis in mammals.

## Cell fate potential of adult neuronal progenitors

Compared to other vertebrate groups, the mammalian parenchyma has higher and more widespread neurogenic potential, but at the same time, markedly reduced regenerative capacities. The key to this apparent paradox lies in the fate of their progeny.

### Cell fate specification during development

The RG fate potential is shaped by spatial and temporal patterning. Morphogen gradients (e.g., BMP, Shh, Wnt) subdivide the VZ in domains of committed progenitors generating specific neuronal subtypes (Jessell, 2000; Puelles et al., 2000; Appan et al., 2023). In the vertebrate telencephalon, pallial RG generate glutamatergic neurons, while sub-pallial RG (e.g., MGE, LGE, CGE, PoA) produce GABAergic neurons (Marin et al., 2000; Cobos et al., 2001; Flames et al., 2007; Luzzati, 2015; Quintana-Urzainqui et al., 2015; Schmitz et al., 2022). RG can also change its potential over time. In the cortex, RG cells sequentially generate deep-layer neurons, upper-layer neurons and then glia (Oberst et al., 2019a; Lin et al., 2021). This sequence is driven at least in part by intrinsic mechanisms, as shown by clonal cultures and was initially thought to be irreversible (Frantz and McConnell, 1996; Shen et al., 2006; Gaspard et al., 2008). However, recent work shows that late RG can resume a previous neurogenic state in response to earlier-stage environmental signals, revealing temporal plasticity (Oberst et al., 2019b). A similar capacity to resume developmental neurogenic sequences has been proposed to support regeneration after lesion in some non-mammalian vertebrate models (Ohnmacht et al., 2016; Zheng et al., 2022; Foucault et al., 2024).

### Fate of adult-generated neurons in non-mammals

In non-mammalian vertebrates, multiple RG domains remain neurogenic throughout life, preserving the embryonic regionalization and commitment (Furlan et al., 2017; Raj et al., 2020). This enables the generation of region-specific neurons and, in some cases, successful brain regeneration (Alunni and Bally-Cuif, 2016; Joven and Simon, 2018; Lust and Tanaka, 2019; Jurisch-Yaksi et al., 2020), as in teleost fishes (e.g., zebrafish and medaka) and urodeles (e.g., axolotl and salamanders) whereas in anurans this capacity is lost upon metamorphosis (Endo et al., 2007; Berg et al., 2011; Kyritsis et al., 2012). In teleost fish, in physiological conditions, newly generated neurons remain mostly confined within their domain of origin and are supposed to acquire region specific identities, although their fate has been verified only in some regions, mainly in the pallium and subpallium (Adolf et al., 2006; Hinsch and Zupanc, 2007; Ganz and Brand, 2016; Lange et al., 2020). In reptiles, besides the olfactory bulb and the medial cortex (homologous to the DG) new neurons are also generated in various telencephalic regions, including the striatum and amygdala (Lopez-Garcia et al., 1988; Font et al., 2001; Delgado-Gonzalez et al., 2011; Grandel and Brand, 2013). In birds, the identity and integration of adult-born neurons have been characterized in greater detail. Within the dorsal pallium, RG cells give rise to different subtypes of glutamatergic projection neurons that integrate into song-related circuits (Nottebohm, 2004; Scott and Lois, 2007). Conversely, subpallial RG cells generate both interneurons that migrate to pallial regions and striatal projection neurons (Scott and Lois, 2007; Kosubek-Langer et al., 2017).

### Fate of adult-generated neurons in mammalian canonical niches

In mammals, SGZ progenitors retain the embryonic commitment of their domain of origin, generating glutamatergic granule neurons throughout life (Bond et al., 2021). In contrast, V-SVZ progenitors, despite originating from multiple pallial and subpallial embryonic domains (Figures 2A,B), converge to produce OB interneurons (Merkle et al., 2007; Dodson et al., 2015; Fuentealba et al., 2015). These interneurons belong to the LGE-MEIS2/PAX6 neuronal class that, during embryonic development, originates mainly from the dorsal lateral ganglionic eminence (LGE; Stenman et al., 2003; Schmitz et al., 2022). With the establishment of the postnatal V-SVZ, this neuron class starts to be produced also by progenitors derived from other embryonic domains (Kriegstein and Alvarez-Buylla, 2009; Bayraktar et al., 2014). In adults, the embryonic regionalization is preserved but repurposed to generate different OB interneuron subtypes (Merkle et al., 2007; 2014). New neurons differentiate but only about half of them integrate into mature circuits (Kempermann et al., 2004; Deshpande et al., 2013; Akers et al., 2014; Sailor et al., 2017; Denoth-Lippuner and Jessberger, 2021). Some progenitors are activated on demand to generate specific OB interneuron subtypes only in specific conditions (Paul et al., 2017; Chaker et al., 2023; 2024).

Heterotopic transplantations indicated that subtype identity depends on the intrinsic commitment of astroglial progenitors (Merkle et al., 2007). Interestingly, these fate choices are only weakly and incompletely discriminated at the transcriptional level, both in the progenitors and in neuroblasts (Tepe et al., 2018; Cebrian-Silla et al., 2021) suggesting similar differentiation trajectories. When the switch to OB interneuron commitment is established remains unclear, but it likely coincides with the gliogenic switch, when the RG begins to generate astrocytes and oligodendrocyte precursors (Fuentealba et al., 2015; Zhang et al., 2020; Lee et al., 2024; Wang et al., 2025). The convergence of diverse progenitor domains toward a single interneuron class likely represents a specific evolutionary adaptation of the mammalian telencephalon, possibly related to the evolution of astrocytes.

### Fate of adult-generated neurons in the mammalian parenchyma

Whether neuronal progenitors retaining embryonic commitments persist in a quiescent state in canonical niches or in the brain parenchyma is still unclear. In some mammalian species, low-level neurogenesis can occur outside the two canonical niches in physiological conditions, particularly in the striatum, where neurogenesis has been proposed to occur also in humans (Bédard et al., 2002; Luzzati et al., 2006; 2014; Ernst et al., 2014; for reviews see Bonfanti and Peretto, 2011; Feliciano et al., 2015; Jurkowski et al., 2020). Brain lesions can further stimulate parenchymal neurogenesis also in the cortex and striatum of rodents (Kernie and Parent, 2010; Magnusson and Frisén, 2016; Williamson et al., 2019). The newborn parenchymal neurons may arise from either the V-SVZ or local progenitors, but their identity and circuit integration remain poorly defined. Early studies proposed that these neurons could adopt appropriate region-specific fates, such as corticospinal neurons after focal cortical ablation (Chen et al., 2004) or medium spiny neurons, the striatal projection neurons, after stroke (Arvidsson et al., 2002), yet such claims have not been subsequently confirmed (Liu et al., 2009; Luzzati et al., 2011; Wei et al., 2011; Magnusson et al., 2014; Nato et al., 2025). Instead, a recurrent and highly consistent observation across independent studies and experimental models is that most newborn parenchymal neurons are short-lived (Gould et al., 2001; Arvidsson et al., 2002; Chen et al., 2004; Luzzati et al., 2006; 2011; Ohira et al., 2010). In line with the neurotrophic theory (Levi-Montalcini, 1987), the physiologic loss of newly generated neurons, reaching up to 50% in adult-born neurons and developing cortical interneurons, is commonly interpreted as a selection process, in which differentiating neurons compete for integration (Kempermann et al., 2004; Denoth-Lippuner and Jessberger, 2021). Similarly, the poor survival of newborn parenchymal neurons has been interpreted as a strong selection, caused by a non-permissive environment. However, transplanted embryonic precursors survive and integrate into the adult parenchyma (Fricker et al., 1999; Wernig et al., 2004) even in the very same environment in which locally generated cells die (Luzzati et al., 2011), suggesting that their survival is regulated by cell-intrinsic factors.

During development, immature neurons can play transient roles in circuit assembly that are unrelated to their functions in mature circuits (Cossart and Garel, 2022). Similarly, in adult niches, immature neurons are endowed with special plastic capacities that affect local circuits before the critical selection windows (Ge et al., 2007; Akers et al., 2014; Sailor et al., 2017). In this view, the initial number of neurons generated may meet the demand of transient plasticity, while selection may subsequently fine-tune this surplus to match the functional requirements of mature circuits. Accordingly, during development, the extent of neuronal selection can range from negligible (Sarma et al., 2011; Gao et al., 2014), to partial, as in the case of interneurons, up to neuron types such as Cajal–Retzius or subplate neurons that, despite integrating into circuits, are almost entirely eliminated by the end of development (Cocas et al., 2016; Riva et al., 2019; Molnár et al., 2020; Elorriaga et al., 2023). In adults, pregnancy induced OB interneurons have a transient life that is dependent on the presence of pups (Chaker et al., 2023).

To explore whether neurons generated by striatal astrocytes after QA lesion correspond to a transient type, we recently combined morphological, functional, connectivity and transcriptomic analyses (Nato et al., 2025). Despite their transient life, these lesion-induced neurons mature morphologically and functionally, integrating into circuits. Single-cell RNA-seq revealed that they do not belong to striatal neuron lineages but rather to the same LGE-MEIS2/PAX6 class as the OB interneurons generated in the V-SVZ. Re-analysis of neurons generated by striatal and cortical astrocytes after Notch abrogation (Magnusson et al., 2020; Zamboni et al., 2020) revealed a widespread commitment of telencephalic astrocytes towards this neuron class. Although this class was thought to contribute only to OB interneurons, in primates, they were recently shown to transiently populate the embryonic striatum and cortex (Schmitz et al., 2022; Wang et al., 2025). Spatial transcriptomics revealed that these cells are also present in the mouse cortex and striatum during embryonic and postnatal development (Nato et al., 2025). A more detailed analysis using Sp8, a specific marker of these cells in the striatum, showed that they are transiently populating the rostro medial striatum up to the weaning period. In this same developmental stage, similar transient Sp8+ cells are present in the lateral striatum of the guinea pig (Figure 4; Luzzati et al., 2014). Striatal LGE-MEIS2/PAX6 cells may thus represent a new developmental player reused in a context-specific manner to support plasticity.

## Conclusions

Like Superman and Clark Kent, astrocytes and NSCs represent two reversible states of the same lineage, and should therefore be viewed as a single cell type (Tasic, 2018; Arendt et al., 2019). This lineage comprises multiple subtypes with partially distinct evolutionary origins and embryonic inductors but converges on the expression of shared gene modules. In mammals, parenchymal astrocytes achieved their highest homeostatic complexity, likely at the expense of their propensity to transit to a neurogenic state. Yet, when reactivated, they faithfully recapitulate the cellular and molecular programs of the ancestral epithelial niches. In these niches, RG cells are tightly interconnected while maintaining extensive contact with neurons and other parenchymal cells through their basal processes. Parenchymal astrocytes rearrange their somata but they preserve tiling, intercellular communication, and neuron–glia crosstalk, effectively transforming the planar epithelial arrangement into a three-dimensional lattice that maintains the ancestral topological relationships. Paraphrasing Eleine Fuchs, astrocytes are architects of their niches (Fuchs and Blau, 2020).

While this expanded distribution of astrocytes allows more localized and specialized neuron-glia interactions, evolution comes with its scars (Krogman, 1951): the widespread latent neurogenic potential may have increased the brain’s vulnerability to cancer. Glioblastoma can reactivate genetic programs of NSCs or early astrogliogenesis, occupying an intermediate state between neurogenic and homeostatic astrocytes (Guo et al., 2023; Sojka et al., 2025; Wang et al., 2025). Some of the mechanisms that sustain neurogenic activation in parenchymal astrocytes may therefore be reused by cancer cells to fuel their growth. In parallel, cellular and molecular mechanisms may have evolved to counterbalance this risk. Deciphering these programs and reconstructing their evolutionary origins may thus reveal novel targets to treat this devastating disease.

In addition, this latent potential could be exploited for regenerative medicine. Multiple attempts have aimed to drive neuronal differentiation in parenchymal astrocytes, typically by inducing forced overexpression of neurogenic factors such as *Sox2* or *Ascl1* (Niu et al., 2013; Leaman et al., 2022; Marichal et al., 2024). These factors, however, act within the shared components of neuronal developmental programs, and are thus expected to trigger only the intrinsic endogenous cell fate potential of the targeted cells (Herrero-Navarro et al., 2021).

While the overall spatial patterning is highly conserved across metazoans, particularly in the nervous system, specific developmental programs can be co-opted and reactivated in different domains, a mechanism also proposed to underlie evolutionary changes in regional neuronal composition (Luzzati, 2015). This plasticity is likely facilitated by the modular organization of cell type-specific regulatory complexes, which rely on a limited set of selector genes (Arendt et al., 2016). Decoding this regulatory logic could enable redirection of the brain’s latent neurogenic potential toward region-appropriate neuronal types. The recent evolutionary shift in adult NSC commitment, together with their epigenetic memory of developmental domains, may facilitate such reprogramming.

Mammals have thus transformed much of their telencephalic neurogenic potential from a reservoir of regeneration to a reservoir of plasticity. Paraphrasing Ramón y Cajal, it will be for the science of the future to change, if possible, this harsh evolutionary choice.
